# Large-Scale S. aureus Screening with Molecular Epidemiology; the Role of MSSA and Community MRSA in Hospital Transmissions

**DOI:** 10.1017/ash.2024.115

**Published:** 2024-09-16

**Authors:** Courtney Takats, Gregory Putzel, Drew Siskin, Alejandro Pironti, Bo Shopsin, Sarah Hochman

**Affiliations:** Emory University; NYU Langone Medical Center; NYU Langone Health

## Abstract

**Background:** The frequency of Staphylococcus aureus transmission in hospitals is unknown: symptomatic infection may occur months after transmission and colonization, and infection prevention efforts rely on indirect measurements, rather than direct detection of transmission events. We implemented a hospital-based S. aureus screening program, combined with whole genome sequencing of S. aureus surveillance and clinical cultures and data extracted from the electronic health record, to identify S. aureus clonal complex-, patient- and location-specific factors associated with S. aureus transmission in our health system. **Methods:** Screening S. aureus cultures were obtained at admission by nasal swab for adults admitted to Medicine, Transplant, Oncology and intensive care, and weekly by swab of nares, axilla and groin for children admitted to intensive care and Oncology at NYU Langone Health in New York City. All methicillin-resistant S. aureus (MRSA) from screening and clinical (blood, wound, sputum) cultures and all methicillin-susceptible S. aureus (MSSA) from screening and blood cultures underwent whole genome sequencing. Isolates from distinct patients with < 2 0 single nucleotide pair differences were considered genetically related. Electronic health data was extracted for descriptive statistics and for spatiotemporal plots to assess plausible transmissions. We used REDCap electronic data capture tools hosted at NYU Grossman School of Medicine and SAS software for data analysis to evaluate S. aureus transmissions between November 2022 and November 2023. **Results:** We analyzed 8,567 S. aureus isolates: including 6,552 screening cultures, 1,008 blood cultures, and 1,007 clinical cultures. We found 424 plausible S. aureus hospital transmissions using sequencing and electronic health data. Screening cultures identified 75% of transmissions that would have otherwise been missed with blood and clinical cultures alone. The majority of positive screening cultures isolated MSSA, but the proportion of transmissions due to MSSA differed by age. In children, MSSA colonization accounted for 62% of transmissions. In adults, only 15% of transmissions were due to MSSA colonization, whereas MRSA colonization accounted for 56% of transmissions. Analysis of adult MRSA isolates by clonal complex found that 45% of transmissions were due to CC8, higher than the 17% among isolates agnostic of transmissions. Emergency departments and the neonatal intensive care unit had the highest number of transmissions. Patients involved in transmissions had longer lengths of stay and frequent hospitalizations. **Conclusions:** A S. aureus screening program, coupled with genome sequencing and electronic health data, can identify patient group, hospital locations and clonal complexes that are at high risk for S. aureus transmissions.

